# An Age-Progression Intervention for Smoking Cessation: A Pilot Study Investigating the Influence of Two Sets of Instructions on Intervention Efficacy

**DOI:** 10.1007/s12529-024-10285-3

**Published:** 2024-05-09

**Authors:** Lucy Walker, Sarah Grogan, Andrew Denovan, Keira Scholtens, Brian McMillan, Mark Conner, Tracy Epton, Christopher J. Armitage, Maria I. Cordero

**Affiliations:** 1 Department of Psychology, Manchester Metropolitan University, Brooks Building, Bonsall Street, Manchester, M15 6GX UK; 2 School of Psychology, Liverpool John Moores University, Liverpool, UK; 3 School of Health and Behavioural Sciences, University of Sunshine Coast, Sunshine Coast, Australia; 4 Centre for Primary Care and Health Services Research, University of Manchester, Manchester, UK; 5 School of Psychology, University of Leeds, Leeds, UK; 6 Division of Psychology and Mental Health, Manchester Centre for Health Psychology, University of Manchester, Manchester, UK; 7 Manchester Centre for Health Psychology, Division of Psychology and Mental HealthNIHR Greater Manchester Patient Safety Research Centre, Manchester University NHS Foundation Trust, Manchester Academic Health Science Centre, University of Manchester, Manchester, UK

**Keywords:** Health behaviour, Smoking cessation, Instructions, Women, Aging

## Abstract

**Background:**

Research on age-progression facial morphing interventions for smoking cessation has not investigated the effect of different instructions for intervention delivery. The objective of this pilot study was to investigate the influence of two instruction types used to deliver the intervention on efficacy of the intervention.

**Method:**

Women were recruited and randomly allocated to an age-progression intervention session with (i) neutral instructions; (ii) instructions designed to reassure; or (iii) a condition that controlled for participant engagement (“control”). The conditions were delivered in a one-time procedure, after which primary (quitting intentions) and secondary (cigarettes/week, quit attempts) outcomes were measured immediately post-intervention, and at 1 and 3 months.

**Results:**

Seventy-two women (*M* = 25.7; SD = 0.9) were recruited and randomly allocated to condition (Neutral* n* = 27, Reassuring *n* = 22, Control *n* = 23). Quitting intentions were higher in the Reassuring versus Control arm (3 months post-intervention, *F* = 4.37, *p* = 0.016, 95% CI [0.231, 2.539], *eta*^2^ = 0.11); quit attempts were greater in the two intervention arms (58%) versus Control (1-month post-intervention, 15%) (*χ*^2^ = 9.83, *p* < 0.05, OR 1.00 [0.28, 3.63]).

**Conclusions:**

Findings highlight the importance of optimising instructions to enhance intervention efficacy.

**Trial Registration:**

clinicaltrials.gov Record: NCT03749382.

**Supplementary Information:**

The online version contains supplementary material available at 10.1007/s12529-024-10285-3.

## Introduction

Smoking is a major cause of illness, death [1], and productivity loss [2]. The World Health Organization (WHO) global action plan (2013–2020) specifies that countries should aim to achieve a tobacco prevalence rate of 19% of the total population by 2025 [3]. Recent health behaviour change research highlights the need to tailor smoking interventions to suit diverse populations [4]. Innovative intervention approaches may help achieve this goal, especially for women, who experience greater difficulty in reducing smoking [5].

Interventions that demonstrate the effect of smoking on facial ageing have previously been shown to be effective in reducing smoking, at least in the relative short-term [6]. Interventions of this nature apply digital aging technology to photos of an individual’s face, in order to demonstrate the combined and progressive effects of aging and smoking on the face. Randomised controlled trials (RCTs) have indicated that age-progression interventions increase quit smoking intentions in comparison to controls [7] and increase smoking abstinence [8]. Qualitative research [9–11] has consistently indicated that participants (particularly women) [9, 11, 12] report being shocked and surprised by the images of smoking on their morphed aged faces, that they are impressed with the intervention, and how the visual illustrations increased their awareness of personal vulnerability to the harmful effects of smoking, and report increased intention to quit following intervention administration.

Although research on age-progression intervention techniques reports positive findings, published studies often lack information on controlling for intervention fidelity, which is a focus for improvement within behaviour change research [13]. One way in which fidelity can be improved is through scripted instruction guides [14]. Previous research has indicated that the language used to deliver task instructions may have a priming effect on the participant, cueing an increased or decreased physiological response [15]. Given the “shocking” impact of the facial morphed images as reported by the participants [11, 12], the language used in instructions could be of special relevance to the efficacy of this type of intervention. In health-based settings, reassuring statements are often provided when delivering health-based news to a patient [16]. Reassurance is understood as the removal of fear or concerns, and reassuring statements are included in a number of clinical guidelines for non-specific conditions [17]. Affective reassurance is introduced with the aim of enhancing practitioner and patient relationships and reducing concerns and fear in the long-term [17]. It could be expected that through implementing language typically used by health practitioners to reduce “shock” and fear, the impact of the intervention on quitting or cutting down cigarettes may vary. Despite this, to the authors’ knowledge, scripted “neutral” instructions alongside delivery of the intervention have only been used in Walker et al.’s (2022) qualitative study where the authors explored women’s accounts of the experience of the intervention [12]. Therefore, the nature of instructions used and impact of specific instruction types (neutral or reassuring) are not well understood in the context of age-progression interventions.

### Aims and Objectives

This pilot study aimed to investigate the influence of two instruction types on efficacy of an age-progression intervention for smoking in women. To address this aim, two sets of instructions were designed: neutral instructions (Neutral) and instructions intended to reassure (Reassuring). The study set out to pilot the impact of both neutral and reassuring verbal instructions, and is the first to investigate the impact of instructions used during the administration of an age-progression intervention in a controlled way. As different instruction types have not previously been tested, hypotheses were developed to guide the pilot inquiry, including (i) participants receiving either of the age-progression intervention arms will have better smoking outcomes in terms of the primary outcome (quit smoking intention) and secondary outcomes (level of smoking behaviour, quit attempts, and abstinence from smoking) at short and long-terms in comparison to the control arm; and (ii) the arm that received reassuring instructions (i.e. designed to reduce the shock impact of the morphed images) will have significantly lower quit smoking intentions than the Neutral arm.

## Method

### Design

**Fig. 1:**
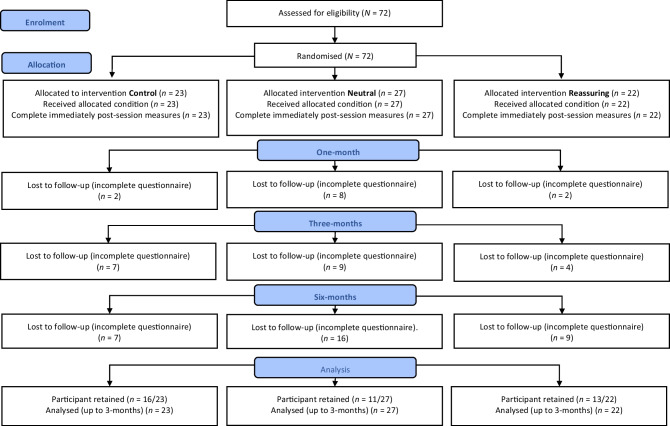
CONSORT flowchart. The number of participants (i) recruited, (ii) allocated to each arm, (iii) who did not complete the questionnaire at each follow-up time point (1, and 3 and 6 months), and (iv) the final number of participants whose data were used in the analysis (excluding 6-month data)

This study used an experimental design with three-arm parallel randomised groups with pre- and post-assessments. Participants were randomised to receive either the age-progression intervention with Neutral or Reassuring instructions, or the active control arm (1:1:1 allocation ratio) on one occasion (see Fig. 1). The results from this pilot will be used to inform subsequent studies.

Comparisons were drawn between either intervention arm and the control, and secondly between the two intervention arms. Previous research has also focused on women under the age of 35 years [7], while the current study extended this age range to 55 years. To ensure randomisation was equally spread for women over 35, stratification (± 35) was implemented. Participants were assessed pre-session (all three conditions), immediately post-session, and at 1, 3, and 6 months post-session administration. Six-month data collection observed a large dropout, so results are not reported here, though are available as supplementary materials (S 1). The protocol was registered prior to data collection on clinicaltrials.gov (Record: NCT03749382). Post-registration, recruitment did not meet the required sample size at the 6-month timepoint; however, due to the novelty of the trial subject, the current results are presented as a pilot study to inform later research.

### Randomisation and Blinding Procedure

A random list of numbers corresponding to one of the three intervention arms was produced using SPSS [18] (V26) and added to sequentially numbered containers separated for ± 35. The research utilised a single blind design within the intervention arms, where participants randomised into either one of the two active intervention arms were blinded as to the instruction type, controlling for bias in follow-up data collection.

### Recruitment and Participants

An estimated sample size of *N* = 101 was based on detecting a medium effect for the primary smoking outcome using post hoc comparison between two groups (*d* = 0.63) as in prior research [7] with alpha level of 0.05 and power = 0.80. Participants were eligible to take part if they were women aged 18–55 years (upper age limit due to the age-progression intervention software parameters) who self-identified as smokers (smoking at least one cigarette a week) and had normal or corrected to normal vision (given the visual aspect of the intervention). Participants were asked to self-exclude if they anticipated sensitivity regarding topics around aging. Recruitment started on 14th of December 2018 and data collection was ceased by the 27th of March 2020; a total sample of 72 women aged 18–54 took part; participants were recruited from university students, staff, and members of local community groups using research adverts, snowballing, and other engagement with community groups. Recruitment and data collection were ceased after this point due to face-to-face data collection restrictions relating to COVID-19.

### Details of Treatment Conditions

#### Age-Progression Intervention

The age-progression facial morphing intervention (APRIL® software, version 2.74) [19] was implemented in two active arms within this trial. The facial-wrinkling effects of smoking demonstrated by the intervention are based on average ageing characteristics taken from a database of 3D scans of smokers. The APRIL® software displayed on a laptop screen worked by taking a photograph of an individual’s face, and considering physical features such as age, ethnicity, and gender, the software illustrated how the person is likely to age up to age 72 years as 2D and 3D images. Brightness and contrast filters were applied as necessary. Facial feature detection points (e.g. mouth, eyes) were manually matched between the participant’s picture and the stock image. The software displayed a time progression of the ageing process on the individual’s photograph, displaying both the smoking and non-smoking images simultaneously side by side on the screen. The intervention administrator guided the participant through the intervention images, using standardised Neutral or Reassuring instructions depending on intervention arm. The intervention duration was approximately 10 min; this included viewing a 2D image on three occasions, followed by a 3D image twice (S2, Fig. 1).

##### Neutral Instructions

Participants randomised to the Neutral arm were delivered the age-progression intervention outlined above with neutral instructions, minimising the influence of the researcher. For example, “please can you close your eyes and open them when I tell you to, you will see your face aged to 72”. These instructions were delivered in a neutral voice with corresponding facial expression (S3).

##### Reassuring Instructions

Participants allocated to receive instructions designed to reassure were delivered the intervention outlined above through reassuring instructions. The instructions followed the same basic instructional statement as in the Neutral Arm, with the addition of reassuring phrases, e.g. “do not be alarmed it is just the morphing process” (S3).

##### Control

**Fig. 2:**
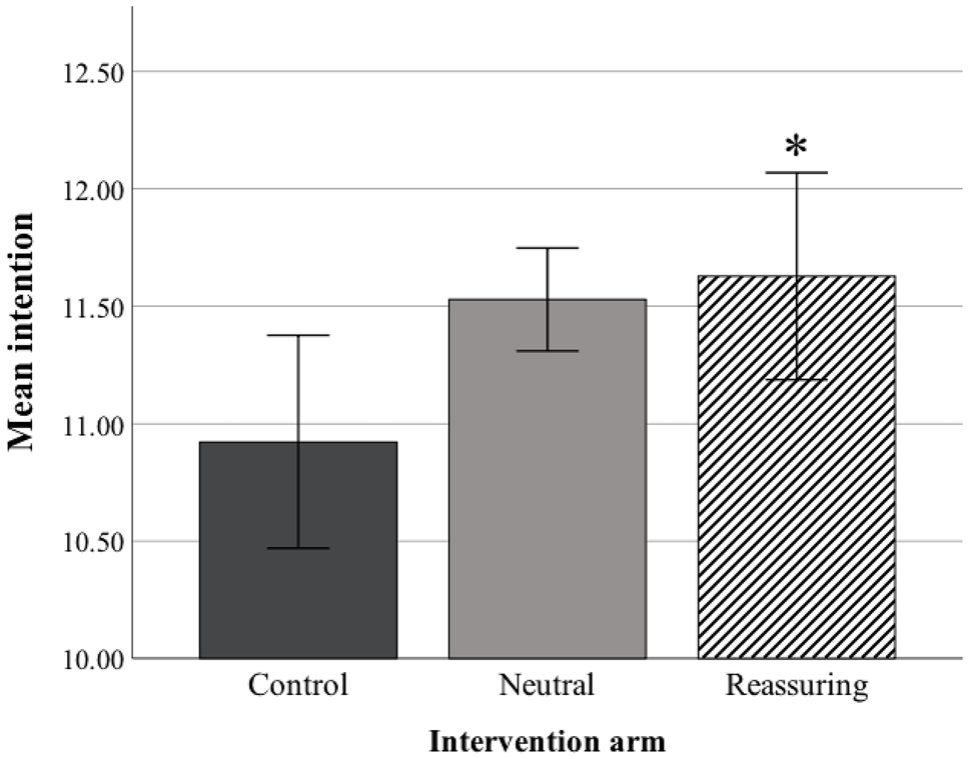
Quit smoking intentions at 3 months post-session bar chart. Bar chart represents mean without baseline covariate. Control = dark grey bar. Neutral = light grey bar, Reassuring = striped bar. Error bars ± 1 SEM. *, *p* < .05 vs Control

An equivalent visual and computer-based control task was developed based on a “spot the difference” game. This echoed the intervention, through use of comparison images on a laptop screen. The task did not include images related to smoking, health, or appearance. As in the intervention arm, standard written UK stop smoking advice was given post-task. Five pairs of images were selected to match the five sequences presented in the intervention, which took approximately 10 min to complete (S2, Fig. 2). The control task was developed to create an equivalent task in terms of participant engagement, allowing for a controlled comparison of the intervention arm content versus solely standard stop smoking written advice.

### Procedure

Test sessions were scheduled as soon as possible after people expressed interest in participating. The sessions took place in controlled environments including Manchester Metropolitan University psychology labs, while all follow-up questionnaires were administered online. Participants were provided with full details regarding the study and given the opportunity to ask questions. The first author obtained informed consent and attached non-invasive physiological sensors to two fingers on the non-dominant hand (for secondary study objectives, not reported here). The initial questionnaire portfolio was then completed (consisting of demographic information, pre-session primary and secondary outcome measures, and the further measures outlined above). Next, all participants were asked to complete a 2-min baseline relaxation period for the purpose of physiological measurement, and participants randomised to the Neutral and Reassuring arms were administered the intervention with corresponding randomised instructions. Participants in the control arm were administered the control task. Following the completion of the session, all participants were asked to rate arousal and received a public health leaflet to view (“It’s so much easier since I quit”: Your guide to quitting for good with Smokefree: Order No. 9000A.DH2900207.07/12). Lastly, physiological sensors were removed, and participants were asked to complete the post-session questionnaire which comprised arousal and the primary outcome measures.

At the end of the session, participants were partially debriefed and provided links to stop smoking support information. The post-session questionnaire portfolio including measures of the primary and secondary outcomes and the rest of the measures were administered at 1, 3, and also at 6 months post-session with the addition of a full debrief given to the participants at the end of data collection (following all data collection).

### Primary Outcome

The primary outcome was quit smoking intentions, assessed via a measure of goal intention [20]. Three items made up the subscale: e.g. “I intend not to smoke in the future” measured using a 13-point Likert scale (1 = strongly disagree to 13 = strongly agree) with higher summed scores ranging from 3 to 39 equating to increased positive smoking intentions. The lowest alpha rating was 0.75 at 1 month.

### Secondary Outcomes

Self-reported smoking behaviour was operationalised as the number of cigarettes smoked each day the week prior to testing, summed to create the sum of cigarettes consumed in the past 7 days. After registration, further binary outcome measures were obtained and operationalised from measures of smoking behaviour. These measures included quit attempts between each data collection time point (Yes or No) and presence of 7-day point abstinence at each data collection time point (abstinent or not abstinent).

### Further Measures

Additional smoking-related measures not reported here included subscales of Ajzen’s theory of planned behaviour [20] (Attitudes, Subjective Norms and Perceived Behavioural Control), and the Fagerstrom Test [21] (Nicotine dependence) was administered (for results see, S1 Table 2) along with other psychological and self-report measures included for secondary objectives of the trial registration.

### Statistical Analysis

All analyses were conducted using SPSS v26. Analysis of the primary and secondary outcome measures was completed using data imputation for dropout using expectation maximisation algorithms [22]. Before imputation, Little’s test and Chi-square tests assessed if data was missing at random (MAR). If data were not MAR, or reached 40% attrition of cases [23], imputation could not be produced. Due to significant data loss at the 6-month data collection time point, imputation was not achieved, so analysis of this time point is not presented below (though data are available in S1, Table 1).

**Table 1:** Sample characteristics pre-intervention

Demographic	Control	Neutral	Reassuring	Total
	*n* = 23	*n* = 27	*n* = 22	*N* = 72
Age M/ SD (min, max)				
Current age	25.5/1.5 (19, 46)	27.0/1.7 (19, 54)	24.1/1.3 (18, 40)	25.7/7.6 (18, 54)
Age starting smoking	17.8/0.6 (15, 26)	17.0/0.5 (13, 22)	16.6/0.4 (11, 20)	17.1/2.4 (11, 26)
Years smoking	7.71/7.87 (0,30)	10.01, 9.56 (0,35)	7.55, 5.83 (0. 24)	8.52, 8.00 (0,35)
Amount usually smoked per day (%)				
1–5	52	48	59	53
6–10	26	41	32	33
11–15	9	11	0	7
16–20	4	0	9	4
21–25	9	0	0	3
Education (%)				
Secondary	0	0	5	1
Further	30	15	13	20
Undergraduate	48	55	59	54
Post-graduate	22	30	23	25
Ethnicity (%)				
White	74	85	100	86
Asian	18	4	0	7
Mixed/multiple ethnic groups	4	7	0	4
Other ethnic group	4	4	0	3

*%* percentage of participants in arm/whole sample, *M* mean, *SD* standard deviation

Differences between intervention arms at each data collection time point (immediately post-session, and 1 and 3 months post-session) on the primary (intentions) and continuous secondary (sum of cigarettes) outcome measures were examined using analysis of covariance (ANCOVA), controlling for respective baseline measures, followed by post hoc tests of difference for significant tests, assessing differences between control and intervention arms and secondly differences between intervention arms. Chi-square tests were conducted for binary secondary outcome variables (quit attempts), at each follow-up time period using only collected data. Sphericity was not assumed and Greenhouse-Giesser corrections were applied for ANOVA-based analysis. Results were adjusted for multiple comparisons (Bonferroni) and for alpha the level used was 0.05.

## Results

### Participant Retention

The number of participants retained at each time point is illustrated in Fig. 1. At 1 month, 83% of women (*n* = 60/72) were retained and at 3 months this dropped to 72% (*n* = 52/72). At 6 months, 56% of participants were retained (*n* = 40/72). Dropout of participants was not significantly different across all intervention arms at 1 (*χ*^2^(2) = 5.23, *p* = 0.073) or 3 (*χ*^2^(2) = 1.51, *p* = 0.471) months. Little’s tests were conducted to check if the data was MAR. At 1 month data was MCAR (*χ*^2^ = 0.10, *p* = 0.751) and at 3 months post-session Little’s test indicated the data was not MCAR (*χ*^2^ = 0.00, *p* = 1.00), but can be considered as MAR. Lastly, at 6 months dropout exceeded 40%. Therefore, maximum likelihood imputation was used up to 3 months post intervention.

### Participant Characteristics

A total of 72 participants were recruited with a mean age of 25.7 years (*SD* = 7.6) with a range of 18–54 years old. Participants on average started smoking at 17.1 years (*SD* = 2.4) ranging from 11 to 26 years old. Most participants smoked 1–5 cigarettes a day (53%) followed by 6–10 (33%) and in small proportions 11–15 (7%), 16–20 (4%), and 21–25 (3%). One percent of participants obtained only secondary education qualifications, while 20% of participants had further education qualifications. The majority (54%) of participants had obtained some undergraduate education while 25% obtained post-graduate education. To see the full list of demographic characteristics and the spread of characteristics across arms, see Table 1.

### Smoking Outcomes

Differences in the primary (intentions) and secondary (sum of cigarettes) outcomes were assessed between arms using ANCOVA at each follow-up time point (immediately post-session, at 1 and 3 months post-session). Binary secondary outcomes (Quit attempt made (YES/NO) and 7-day point abstinence (Abstinent/Not Abstinent)) were assessed using Chi-square tests at follow-up time points (see Table 2 for results from all trial outcomes across all arms).

**Table 2:** Summary table representing ANCOVA results for primary and secondary outcomes, and Chi-square tests for binary secondary outcomes

Time point	*n*	Control	*n*	Neutral	*n*	Reassuring	*F*	*p*	*eta*^2^
**Primary outcome**									
Intentions (*M, SD*)									
Post-session	23	11.7 (1.6)	27	11.9 (1.3)	22	10.8 (3.0)	0.21	0.812	0.01
1 month	23	11.4 (1.8)	27	11.4 (1.5)	22	11.1 (2.9)	0.60	0.551	0.02
3 months	23	10.9 (2.2)	27	11.5 (1.1)	22	11.6 (2.1)	4.37*	0.016	0.11
**Secondary outcome**									
Sum of cigarettes (*M, SD*)									
1 month	23	40.2 (30.7)	27	36.0 (20.0)	22	42.3 (42.3)	1.14	0.327	0.03
3 months	23	33.1 (29.4)	27	32.6 (26.0)	22	31.1 (32.6)	0.16	0.854	0.01
**Binary secondary outcome**									
	*n*	Control	*n*	Neutral	*n*	Reassuring	*χ*^2^	OR (95%CI)	
								*N*	*R*
Quit attempt (%)									
1 month	20	15	19	59	19	58	9.83*	0.13 (0.03, 0.59)	1.00 (0.28, 3.63)
3 months	16	44	19	68	18	56	2.16	0.62 (0.16, 2.42)	1.73 (0.45, 6.63)
7-day point abstinence (%)									
1 month	21	5	19	11	20	20	2.41	0.20 (0.02, 1.97)	0.44 (0.07, 2.76)
3 months	16	6	19	21	19	26	2.44	0.19 (0.02, 1.80)	0.75 (0.17, 3.36)

Analysis of covariance (*ANCOVA*) with baseline values as covariates, Degrees of freedom (*df*), 1,68 *η*_*p*_^*2*^, partial eta squared, *%*, percentage of participants made quit attempt/abstinent within arm, *OR* (odds ratio): reference category control arm, *N* Neutral,* R* Reassuring

*p < 0.05

A between-participants effect of the intervention arm was observed on quit smoking intentions at 3 months post-session *F*_(1,68)_ = 4.37, *p* = 0.016, *η*_*p*_^2^ = 0.11 (Table 2), with a medium effect size. Post hoc tests indicated that quit smoking intentions were higher in the Reassuring arm (*M* = 12.1) compared to the control (*M* = 10.7) (*p* = 0.013, 95% CI [0.231, 2.539]) (differences in quit smoking intentions are depicted in Fig. 2).

At 1-month post-session, there was a significant difference in the presence of a quit attempt. Specifically, 15% (*n* = 3/20) of control participants reported a quit attempt compared to 58% (*n* = 11/19) in both the Neutral and Reassuring arms.

## Discussion

The current study provides a pilot investigation of two standardised instructions (Neutral and Reassuring) for the delivery of an age-progression intervention for smoking in women.

As expected, we found that the intervention prompted spontaneous quit attempts at 1 month post-intervention, as women within both intervention arms reported more instances of quit attempts in comparison to the Control arm at this point. In addition, the Reassuring arm (but not the Neutral arm) had significantly greater effects on quit smoking intentions at the 3-month timepoint than those in the Control arm. Findings indicate that both instruction types create some positive effects on smoking in comparison to the control arm whereas the Reassuring instructions created a more sustained impact on the primary outcome.

Regarding the effect of the facial morphing intervention on smoking intentions, our findings replicated those reported in a previous trial with only the intervention and control condition [7, 8], in which quit smoking intentions were increased in comparison to controls. Unlike in previous reports [7], this effect was observed at the longitudinal timepoint of 3 months rather than the immediate post-session measure. Levels of intentions could have been raised in the Reassuring arm due to the catalyst of the intervention administration, reaching significance only at the 3-month post-intervention timepoints. Whereas in previous research an immediate effect was observed, no sustained difference in smoking outcomes was observed [7].

In addition to intentions, behaviour in the form of smoking abstinence was also evidenced more frequently within the Reassuring arm. A limitation highlighted in previous investigations of age-progression interventions is the presence of an intention-behaviour gap [24] in which only intent to change the behaviour or cognitive activation of the threat was observed, rather than the behaviour itself. Bridging this gap, i.e. changing both intentions and behaviours, is key to the success of behavioural interventions [25]. In addition to intentions, behaviour in the form of quit smoking attempts was evidenced within the Reassuring arm compared to the control, in a similar proportion of participants as in previous research intervention arms [8]. Interestingly, the current trial evidences both changes in behaviours and intentions when delivered via Reassuring instructions, across 3 months post intervention. No significant effect was observed when the intervention was delivered via Neutral instructions compared to the control at this later timepoint. The results may indicate that the intervention delivered using instructions intended to reassure led to stronger cognitive activation processes, whereby the condition had greater impact on the appraisal of smoking threat leading to changes in smoking behaviour [26].

The current research is the first of its kind to successfully pilot the implementation of different sets of scripted instructions alongside the age-progression intervention. Results show promise as to the effectiveness of instructions with reassuring elements; however, the mechanism of effect of these instructions remains unclear. Providing verbal reassurance has previously been discussed in the context of medical assessments, and is indicated to have two classes of verbal cues which include emotional reassurance and secondly reassurance as to the absence of the relevant condition [16]. This emotional reassurance is suggested to reduce fear and stress [16], while other research [27] within medical field suggests by providing verbal reassurance proactive healthy behaviours are encouraged. The intervention has previously been indicated to create high levels of fear [10–12], which could induce avoidance to the intervention message [28]. Therefore, through reassurance stress could be reduced and intervention efficacy increased.

However, others indicate that reassurance may have a paradoxical effect in which fear is increased [29]. Consequently, the emotionally reassuring statements used for the Reassuring intervention instructions could have induced an increased level of fear within participants. This suggested paradoxical effect of the Reassuring instructions aligns with the Ironic Process Theory [30] that proposes that deliberate attempts to supress certain thoughts could make them more likely to appear, especially under conditions that reduce cognitive capacity (such as under stress). Closa Leon and colleagues [15] support this theory as participants who received supportive and reassuring instructions prior to an anger stress-inducing task displayed increased cardiac output suggestive of an increased stress response. Within the current trial, if the introduction of reassuring statements induced mild stress, participants could be more likely to be persuaded to change their behaviour [31, 32] due to an increase in stress-induced attentional vigilance (sustained allocation of attention) towards subsequent stop smoking information provided [33]. The current trial has piloted the initial implementation of these instruction types; future research can seek to further investigate this impact on a larger sample and measure levels of stress induced by instruction types in order to understand the mechanism of effect. Future research should also explore the underlying mechanisms of this effect through focusing on measures of attitudes alongside physiological measures of stress.

Overall, the pilot findings suggest that the use of Reassuring instructions when delivering the age-progression intervention may enhance the impact of the intervention on smoking behaviors. This finding is highly relevant especially when comparing efficacy of the intervention between prior studies. Previous research investigating the efficacy of a facial morphing intervention for smoking cessation did not implement, or at least failed to report, the use of scripted instructions that could have contributed to the differences in the intervention efficacy reported [7, 8]. The use of scripted instructions would aid the fidelity of this intervention type, plus aid the reporting of health behaviour change interventions and increasing validity of findings [13]. Therefore, in order to develop a strong evidence base, the use of scripted instructions as in this pilot should be the precedent for age-progression intervention research going forward and form the basis of larger sample size trials.

### Strengths and Limitations

A main strength of the trial was the closely comparable active control (missing from many previous RCT studies in the area). In previous investigations of appearance-based interventions [7, 8], researchers spent a longer duration with the participants in the intervention arm which may have potentially confounding effects on the findings as better outcomes may be associated with increased interaction with the researcher [34]. The current research has controlled for this factor through equivalent timing of arms. Furthermore, other strengths of the research include the stratified randomisation and long-term longitudinal data collection. Hence, the current pilot serves to advance research investigating both age-progression and brief general smoking cessation interventions through the rigorous methods employed.

A limitation of the study was the loss of participants at the 6-month post timepoint preventing reliable analysis of the results at this time. It is also worth noting that COVID-19 restrictions impacted both recruitment and follow-up data collection, as 30% of the 6-month follow-up responses were due to occur after the initial UK COVID-19 lockdown order, which may have influenced levels of attrition at this time. Imputation of missing data was conducted up to 3 months post intervention following recommended statistical procedures [22]; however, caution should be applied in interpreting findings. Future research should seek to implement additional retention strategies at this point, though this was not possible here for reasons explained above. Furthermore, although physiological measurements and procedures that were implemented for the wider study were minimal, they had the potential to raise stress in participants. This was however minimised using a relaxation period prior to intervention delivery.

### Implications

Age-progression techniques have been gaining momentum for a number of years [6, 12, 35], with specific focus on how the intervention approach can benefit women [11, 12]. The findings of this pilot suggest that the efficacy of these techniques could be further increased by implementing scripted instructions which are intended to reassure the participant throughout the experience. However, further research is needed to confirm these findings. Researchers adopting scripted instructions into their intervention work should be careful to adhere exactly to their scripts. Researchers should also control for other variables as far as possible, such as who delivers the intervention and the experimental setting, in order to avoid these factors impacting on levels of stress and arousal, so that the focus remains on the intervention and instruction content. The importance of consistency in intervention delivery, as highlighted here, has implications for a range of intervention-based research, as variations in the way the intervention is delivered could affect their success, as arousal created due to instruction content could influence intervention acceptance and behaviour change [31, 33].

## Conclusions

The current study pilot study tested the introduction of both neutral and reassuring instructions alongside an age-progression intervention for smoking. Conclusions that can be drawn are (1) instructions designed to reassure participants during intervention delivery increased quit smoking intentions at 3 months post-session; (2) reassuring instructions could be beneficial for the efficacy of the intervention; (3) through piloting the intervention and instruction types, results support the need for larger scale trials, in order to confirm findings and investigate further the underlying mechanism of effect.

## Supplementary Information

Below is the link to the electronic supplementary material.

Supplementary file1 (DOCX 20 KB)

Supplementary file2 (DOCX 251 KB)

Supplementary file3 (DOCX 14 KB)

## Data Availability

The authors are open to data sharing of de-identified data collected. Application, with rationale, may be made to corresponding author at Manchester Metropolitan University, lucy.walker@mmu.ac.uk.
