# Supplementary material for: An Age-Progression Intervention for Smoking Cessation: A Pilot Study Investigating the Influence of Two Sets of Instructions on Intervention Efficacy
Source: Int J Behav Med. 2024 May 9;33(1):128–37. doi: 10.1007/s12529-024-10285-3 (PMC12935819; doi:10.1007/s12529-024-10285-3)
Supplement: Supplementary file 1 — Supplementary file1 (DOCX 20 KB) [file 12529_2024_10285_MOESM1_ESM.docx]

***Supplementary file 1***

***Table 1.****Summary table representing ANCOVA results for primary and secondary outcome, and Chi square tests for binary secondary outcomes for 6-months post measure*

| Time point | *n* | Control | *n* | Neutral | *n* | Reassuring | *F* | *p* | *eta^2^* | |
| --- | --- | --- | --- | --- | --- | --- | --- | --- | --- | --- |
| **Primary outcome** (*M, SD*) | | |  | | | |  |  | | |
| Intentions | 16 | 10.5 (2.8) | 11 | 11.0 (2.7) | 13 | 11.9 (2.6) | 2.43 | .102 | .12 | |
| **Secondary outcome** (*M, SD*) | | |  | | | |  |  | | |
| Sum of cigarettes | 16 | 37.7 (37.9) | 11 | 35.8 (25.9) | 13 | 23.0 (33.3) | 1.94 | .159 | .10 | |
| **Binary secondary outcome** (%) | | | | |  |  |  |  | | |
|  | *n* | Control | *n* | Neutral | *n* | Reassuring | χ^2^ | OR (95 %CI) | | |
|  |  |  |  |  |  |  |  | N | | R |
| Quit attempt | 15 | 33 | 11 | 45 | 13 | 54 | 1.55 | .39  (.09, 1.78) | | .71 (.14, 3.58) |
| 7 day point abstinence | 16 | 0 | 11 | 9 | 13 | 31 | 6.37* | .00 (.00) | | .23 (.02, 2.41) |

*Note: Analysis of covariance (ANCOVA) with baseline values as covariates. degrees of freedom (df) = 1,68, η_p_^2^ = partial eta squared, % = percentage of participants made quit attempt/abstinent within arm. OR (odds ratio): reference category control arm, N = Neutral, R = Reassuring *, p<0.05*

***Table 2.*** *Summary table representing ANCOVA results for additional secondary outcomes.*

| Time point | *n* | Control | | *n* | Neutral | *n* | Additional | *F* | *p* | *eta^2^* |
| --- | --- | --- | --- | --- | --- | --- | --- | --- | --- | --- |
| Attitudes (*M, SD*) | | |  | | | | |  |  |  |
| post-intervention | 23 | 11.3 (1.7) | | 27 | 10.7 (2.8) | 22 | 10.7 (2.5) | .09 | .822 | .01 |
| 1-month | 23 | 11.4 (1.8) | | 27 | 11.4 (1.5) | 22 | 11.1 (2.9) | .11 | .895 | .00 |
| 3-months | 23 | 11.0 (1.7) | | 27 | 11.4 (1.5) | 22 | 10.8 (2.6) | .83 | .441 | .02 |
| 6-months | 16 | 11.6 (1.4) | | 11 | 10.5 (2.7) | 13 | 12.0 (1.8) | 1.95 | .157 | .10 |
| PBC (*M, SD*) | | |  | | | | |  |  |  |
| post-intervention | 23 | 9.6 (2.5) | | 27 | 8.9 (2.3) | 22 | 9.1 (2.8) | .09 | .917 | .00 |
| 1-month | 23 | 9.4 (2.3) | | 27 | 8.7 (2.3) | 22 | 8.6 (2.9) | .37 | .692 | .01 |
| 3-months | 23 | 9.5 (2.6) | | 27 | 8.2 (2.3) | 22 | 9.1 (2.8) | 1.08 | .345 | .03 |
| 6-months | 16 | 9.4 (2.6) | | 11 | 8.5 (2.2) | 13 | 9.3 (3.3) | .40 | .674 | .01 |
| SN (*M, SD)* | | |  | | | | |  |  |  |
| post-intervention | 23 | 2.4 (2.0) | | 27 | 2.2 (1.8) | 22 | 2.0 (1.5) | 1.77 | .178 | .05 |
| 1-month | 23 | 2.1 (1.7) | | 27 | 2.4 (2.0) | 22 | 1.6 (1.1) | 1.91 | .155 | .05 |
| 3-months | 23 | 2.3 (1.9) | | 27 | 2.3 (2.0) | 22 | 1.6 (.7) | 2.00 | .144 | .06 |
| 6-months | 16 | 2.0 (1.9) | | 11 | 2.2 (1.9) | 13 | 1.9 (2.4) | .29 | .752 | .02 |
| Fagerstrom (M,SD) | | | | | | | | | |  |
| 1-month | 23 | 2.05 (1.67) | | 27 | 2.39 (1.99) | 22 | 1.59 (1.06) | 1.16 | .319 | .033 |
| 3-months | 23 | 2.02 (2.22) | | 27 | 1.99 (1.85) | 22 | 1.63 (2.45) | .838 | .452 | .024 |
| 6-months | 16 | 1.63 (1.99) | | 11 | 2.45 (2.16) | 13 | 1.15 (2.03) | 2.27 | .118 | .11 |

*Note: Analysis of covariance (ANCOVA) with baseline values as covariates. degrees of freedom (df) = 1,68, η_p_^2^ = partial eta squared. PBC = perceived behavioural control, SN = subjective norms.*

**, p<.05*
