# Supplementary material for: An Age-Progression Intervention for Smoking Cessation: A Pilot Study Investigating the Influence of Two Sets of Instructions on Intervention Efficacy
Source: Int J Behav Med. 2024 May 9;33(1):128–37. doi: 10.1007/s12529-024-10285-3 (PMC12935819; doi:10.1007/s12529-024-10285-3)
Supplement: Supplementary file 2 — Supplementary file2 (DOCX 251 KB) [file 12529_2024_10285_MOESM2_ESM.docx]

***Supplementary material file 2.***

***Figure 1. Example images from the intervention delivery***


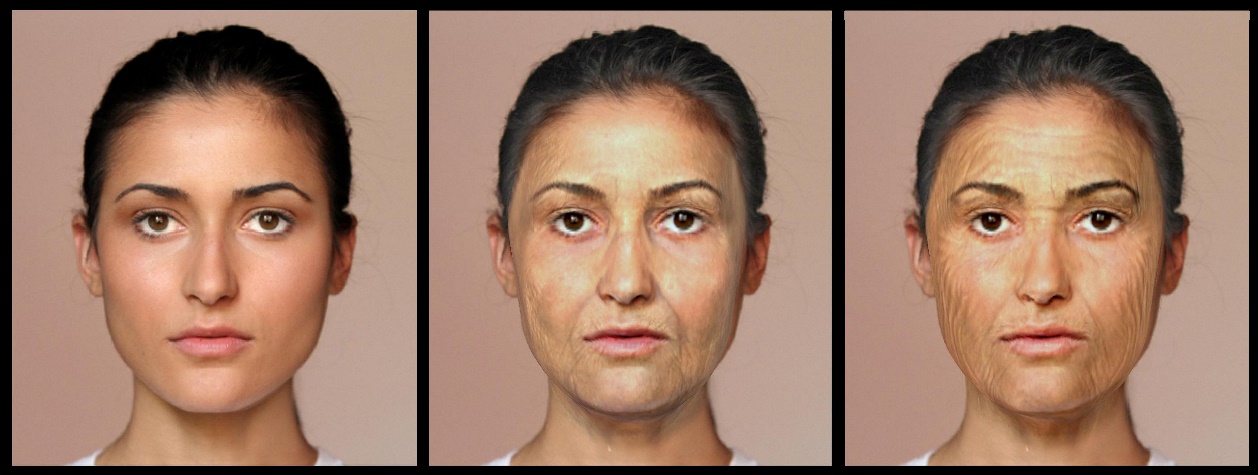


*Note: Photo demonstrates on the far left woman at 22 years, in the middle aged to 72 years without smoking with the AprilAge software and lastly on the right aged up to the age of 72 with the effects of smoking with the Aprilage software.*

***Figure 2. Example images from the control task delivery***


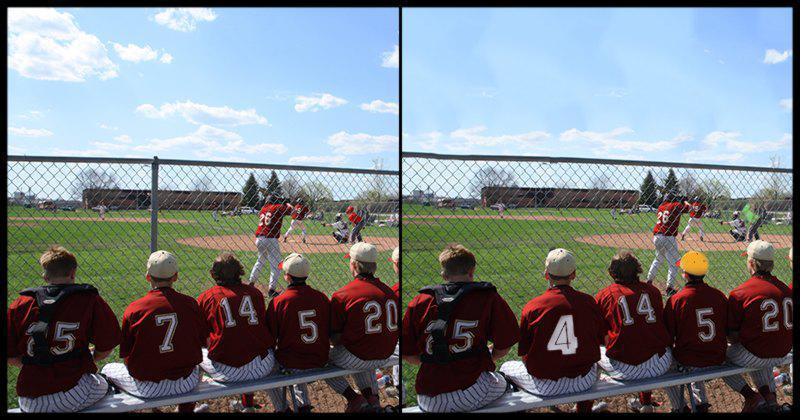


***Note:*** *Example “spot the difference” task image with correct answers*

*Correct answers circled on the right-hand side image. Arrow points to the correct identification of missing clouds in the right-hand image.*

*Images sourced (in line with the fair use of images for educational and research purposes).*
