# Supplementary material for: An Age-Progression Intervention for Smoking Cessation: A Pilot Study Investigating the Influence of Two Sets of Instructions on Intervention Efficacy
Source: Int J Behav Med. 2024 May 9;33(1):128–37. doi: 10.1007/s12529-024-10285-3 (PMC12935819; doi:10.1007/s12529-024-10285-3)
Supplement: Supplementary file 3 — Supplementary file3 (DOCX 14 KB) [file 12529_2024_10285_MOESM3_ESM.docx]

***Intervention instructions.***

**Neutral Instructions.**

I am now going to show you the intervention. (open laptop/tablet, open April or webcam).

I am going to take a picture of your face, please position yourself in the centre of the screen and keep a neutral expression, (for participants with glasses ask to remove).

(load picture in APRIL and fill in set up information) what is your age and ethnicity?

I am now going to edit your picture to match the stock image and match up the points of the face.

(once finished set up) On the screen you will see 2 pictures of your face, both pictures will age up to 72 each time, the one on the left will always be non-smoking and the one on the right will be with the effect of smoking.

*Morph2D* please can you close your eyes and open them when I tell you to. You will see your face aged to 72.

Open your eyes; can you see any differences between the images?

*Morph2D_R -* I am now going to show you the aging process, when I press play you will see both images age to 72. (Press play) can you see any differences?

*Morph2D_R’ -* I am going to repeat the aging process again now (press play) Can you see any other differences?

*Morph3D -*I am now going to change the pictures to a 3D image of your face, I am going to show you again the aging process and after you can move your own face around to view the sides and underneath. (give demonstration), (Press play) can you see any differences?

You can now use the mouse or keypad to move your face around (give demonstration).

*Morph3D_R -* I am going to repeat the aging again in the 3D view (press play) Can you see any more differences? Would you like to move your face around again?

*Participant Lead intervention time-* You can now have a look at the intervention on your own, you can drag the progress bar to different age levels. Notify me when you have finished

**Reassuring Instructions**

I am now going to show you the intervention, it’s quite simple and I will guide you through it. (open laptop/tablet, open April or webcam).

I am going to take a picture of your face, please position yourself in the centre of the screen and keep a neutral expression, (for participants with glasses ask to remove).

(load picture in APRIL and fill in set up information) what is your age and ethnicity?

I am now going to edit your picture to match the stock image and match up the points of the face.

(once finished set up) On the screen you will see 2 pictures of your face, both pictures will age up to 72 each time, the one on the left will always be non-smoking and the one on the right will be with the effect of smoking.

*Morph2D -* please can you close your eyes and open them when I tell you to open them, it can be a bit unexpected, do not be alarmed you will just see your face aged to 72. (smile empathically). Open your eyes, can you see any differences between the images?

It’s normal to be surprised by the effects of time, especially if it’s on our own picture (smile empathically).

*Morph2D_R -* I am now going to show you the aging process, when I press play you will see both images age to 72, again do not be alarmed as it is just the morphing process. (Press play) can you see any differences? (smile empathically).

*Morph2D_R’ -* I am going to repeat the aging process again now, it is exactly the same to what you just saw, so no new surprises (press play) Can you see any other differences?

*Morph3D -*I am now going to change the pictures to a 3D image of your face, I am going to show you again the aging process and after you can move your own face around to view the sides and underneath. (give demonstration), (Press play) can you see any differences? don’t worry it is not that much different from the previous images.

You can now use the mouse or keypad to move your face around (give demonstration).

*Morph3D_R -* I am going to repeat the aging again in the 3D view (press play) Can you see any more differences? Would you like to move your face around again? It’s ok if you have nothing to add.

*Participant Lead intervention time-* You can now have a look at the intervention on your own, you can drag the progress bar to different age levels. Notify me when you have finished
